# In Silico Validation of Personalized Safe Intervals for Carbohydrate Counting Errors

**DOI:** 10.3390/nu15194110

**Published:** 2023-09-22

**Authors:** Débora Amorim, Francisco Miranda, Carlos Abreu

**Affiliations:** 1 ADiT-LAB, Instituto Politécnico de Viana do Castelo, Rua Escola Industrial e Comercial de Nun’Álvares, 4900-347 Viana do Castelo, Portugal; deboram@ipvc.pt; 2 Instituto Politécnico de Viana do Castelo, Rua Escola Industrial e Comercial de Nun’Álvares, 4900-347 Viana do Castelo, Portugal; fmiranda@estg.ipvc.pt; 3 Center for Research and Development in Mathematics and Applications (CIDMA), Department of Mathematics, University of Aveiro, 3810-193 Aveiro, Portugal; 4 proMetheus, Instituto Politécnico de Viana do Castelo, Rua Escola Industrial e Comercial de Nun’Álvares, 4900-347 Viana do Castelo, Portugal; 5 Center for MicroElectroMechanical Systems (CMEMS-UMINHO), University of Minho, Campus Azurém, 4800-058 Guimarães, Portugal

**Keywords:** type 1 diabetes mellitus, insulin therapy, personalized medicine, carbohydrate counting errors

## Abstract

For patients with Type 1 diabetes mellitus (T1DM), accurate carbohydrate counting (CC) is essential for successful blood glucose regulation. Unfortunately, mistakes are common and may lead to an incorrect dosage of prandial insulin. In this work, we aim to demonstrate that each person has their own limits for CC errors, which can be computed using patient-specific data. To validate the proposed method, we tested it using several scenarios to investigate the effect of different CC errors on postprandial blood glucose. Virtual subjects from the T1DM Simulator were used in a clinical trial involving 450 meals over 90 days, all following the same daily meal plan but with different intervals for CC errors near, below, and above the limit computed for each patient. The results show that CC errors within personalized limits led to acceptable postprandial glycemic fluctuations. In contrast, experiments where 50% and 97.5% of the meals present a CC error outside the computed safe interval revealed a pronounced degradation of the time in range. Given these results, we consider the proposed method for obtaining personalized limits for CC errors an excellent starting point for an initial assessment of patients’ capabilities in CC and to provide appropriate ongoing education.

## 1. Introduction

Type 1 diabetes mellitus (T1DM) is a chronic autoimmune disease characterized by the destruction of pancreatic β-cells, responsible for insulin production. T1DM typically develops in childhood or early adolescence but can occur at any age. Unlike Type 2 diabetes, T1DM is not associated with lifestyle factors. While the pathogenesis of T1DM is not fully understood, it is believed to involve a combination of genetic and environmental factors. In T1DM, the immune system mistakenly targets and destroys the β-cells which leads to dysglycemia and makes the patients insulin-dependent [1,2]. Dysglycemia refers to fluctuations in blood glucose (BG) outside the safe range, considered between 70 and 180 mg/dL by the American Diabetes Association (ADA) and the European Association for the Study of Diabetes (EASD) [3]. Hypoglycemia occurs when BG levels are below the safe range, causing acute complications such as confusion, tachycardia, shakiness, and irritability [3]. Moreover, severe hypoglycemia may lead to behavioral changes, visual disturbances, seizures, a loss of consciousness, coma, or even death. Hypoglycemic events involving loss of consciousness or requiring the assistance of another person may be frightening and have a substantial negative effect on the quality of life of patients and their families [4]. On the other hand, BG levels above the safe range, hyperglycemia, lead to long-term complications such as retinopathy, nephropathy, and cardiovascular disease [5]. Severe hyperglycemia symptoms include polyuria, polydipsia, and weight loss, but unlike hypoglycemia, life-threatening complications associated with hyperglycemia events are not imminent but long-term, so patients fear more hypoglycemia [4,6]. Although, most of the morbidity and mortality associated with diabetes are related to complications derived from chronic hyperglycemia.

To avoid such complications, patients with T1DM are treated using insulin replacement regimens, requiring basal insulin and mealtime insulin to compensate for the meals’ carbohydrates (CHO) content [7]. Carbohydrate counting (CC) is widely recommended as a meal-planning tool for T1DM patients. Using this approach, patients need to dose prandial insulin according to Equation (1) [8].

(1) $$B=\frac{CHO}{ICR}+\frac{G-G_{T}}{ISF}-IOB,$$

where B is the bolus insulin in units (U), CHO is the quantity of carbohydrates to be consumed in g, ICR is the insulin-to-carbohydrate ratio in g/U, G is the preprandial BG and G_T_ is the target BG in mg/dL, ISF is the Insulin Sensitivity Factor in mg/dL/U, and IOB (Insulin on Board) is the insulin remaining active from the previous bolus administrations in U. In CHO, the patient introduces the carbohydrate intake planned for each meal resulting from their estimation by performing CC.

Thus, accurately performing CC is paramount to dose the insulin bolus and, consequently, to achieve proper BG regulation [9,10]. Indeed, CHO over- and underestimations introduce errors in the bolus insulin that may lead to hypoglycemic and hyperglycemic episodes, respectively. Therefore, CC demands nutritional education and training, which can be challenging for most individuals [11]. Several studies corroborate that T1DM patients frequently commit errors in CC [9,12,13,14,15,16]. In addition, CC errors proportionally relate to the size of meals, with underestimations being more common in large meals, which can result from fearing hypoglycemic events once the consequences could be immediate [12,15,16,17]. Smart, in [18,19], assessed the impact of different CHO estimation errors on postprandial glycemia, concluding that errors around 20 g in meals containing 60 g of CHO may lead to undesirable BG excursions, while variations around 10 g result in acceptable BG fluctuations.

Nevertheless, each individual has their own characteristics, such as ICR and ISF, so these generalized limits provide orientation but can be improved. For that purpose, Abreu et al., in [20], presented an analytic method to determine the safe limit to CC error using personalized data. The authors used Equation (2) to calculate the maximum admissible absolute error while counting carbohydrates to ensure the postprandial BG level was on target.

(2) $${∆CHO}_{max}=\frac{ICR}{ISF}\cdot min\{G_{H}-G_{T},G_{T}-G_{L}\},$$

where ∆CHO_max_ is the maximum absolute error allowed when performing CC; G_H_ and G_L_ are the high and low BG thresholds, respectively, for maintaining BG levels within range.

Based on patient-specific CC maximum errors, personalized CC educational programs can be tailored to address the needs of each patient rather than following a one-size-fits-all approach. In this way, a patient with a maximum allowable error of less than the general recommendation of 10 g should be made aware and trained more rigorously, while patients with a higher margin of error can feel more confident in performing CC and improving their treatment adherence. Indeed, knowing patients’ specific limits to CC error is crucial to improve their motivation and engagement.

To the best of our knowledge, apart from the one proposed by Abreu et al. [20], no other method allows computing patient-specific limits for the CC error. Therefore, it is crucial to assess its effectiveness. The main goal of this work was to study if the upper limit obtained from Equation (2) could be taken as a boundary value to preserve on-target postprandial BG. We aimed to understand the impact of different CC errors within and outside the safe limits in patients’ glycemic behavior. Additionally, we intended to analyze the differences between distinct profiles—patients with a tendency to overestimate CHO, patients who underestimate, and patients who used to perform both over- and underestimations of CHO. To achieve this goal, an in silico trial was designed using the University of Virginia (UVA)/Padova Type 1 Diabetes Mellitus Simulator (T1DMS).

## 2. Methods

To evaluate the effectiveness of Equation (2), we conducted a series of in silico trials using the T1DMS approved by the Food and Drug Administration, which allows the evaluation of 33 virtual patients under multiple scenarios. Our study involved several experiments with different CHO estimation errors, both within and outside the calculated safe range for each patient. Subsequently, we compared the glycemic data obtained from these scenarios with the data collected in a control scenario, where there was no discrepancy between the actual CHO content and the patients’ estimations.

### 2.1. Subjects

This study encompassed all the subjects available on the T1DMS, which includes 11 adults, 11 adolescents, and 11 children. The different age groups presented considerable differences in the ICR and the ISF. The adult population had a mean ICR of 15.9 ± 4.5 g/U and an ISF of 42.2 ± 8.0 mg/dL/U, whereas the adolescents exhibited an ICR of 17.6 ± 7.0 g/U and an ISF of 57.1 ± 13.8 mg/dL/U. The children displayed an even more significant difference, with an ICR of 26.5 ± 5.3 g/U and an ISF of 117.8 ± 27.7 mg/dL/U. The ICR and ISF values of each patient were used in Equation (2), along with the glucose parameters G_L_, G_T_, and G_H_ defined as 70, 100, and 180 mg/dL, respectively, following the ADA and EASD guidelines [3]. By carrying this out, per-subject safe limits for the error on the CHO estimations were computed, as presented in Table 1. The ∆CHO_max_ values for all subjects had a mean value of 9.13 ± 2.99 g, with a maximum of 16.55 g and a minimum of 5.37 g.

### 2.2. Simulation Scenarios

All the simulations conducted were open-loop, covering 90 days, with patients undergoing intensive insulin therapy using multiple daily injections following a basal-bolus scheme. The T1DMS automatically determined the basal rate based on the subject-specific optimal rate. The bolus insulin for each meal was calculated using Equation (1), with the ICR and ISF of each patient set as previously mentioned, the GT established at 100 mg/dL, and the IOB assumed to be negligible, as the meal plan presented in Table 2 was designed to ensure proper time intervals between meals [8]. Equation (3) provides the CHO values used in Equation (1), representing the estimated amount of CHO in each meal while accounting for potential CC errors.

(3) $$CHO={CHO}_{True}-∆CHO,$$

where CHO_True_ is the real quantity of CHO in the meal, according to the meal scheme presented in Table 2, and ∆CHO∼N (µ, σ^2^) is a uniformly distributed random variable representing the CHO estimation error.

From the meal plan in Table 2 and using different ∆CHO distributions, nine scenarios were designed with different error ranges. These errors will affect the amount of bolus insulin administered in all 450 meals. An error-free scenario was developed as the control experiment (E_0_), where patients eat and dose the bolus insulin according to the nutritional plan without estimation errors. All other experiments aimed to observe the impact in the postprandial BG of different CC errors, i.e., near, below, and above the maximum allowed for each patient. Moreover, to better represent real possible error trends in patients when estimating the amount of CHO, the experiments encompassed symmetric and polarized error intervals. In this way, we could analyze the impact of errors on various patient profiles, including those who consistently overestimate CHO content, those who consistently underestimate it, as well as those who make mistakes above and below the correct CHO content. To evaluate the impact of CC errors in patients with the same tendency to CHO underestimation and overestimation, we designed experiment E_1_, whose ∆CHO distribution was centered on zero and generated errors within ±∆CHO_max_ with a confidence interval (CI) of 95%.

The remaining experiments aimed to evaluate the effect of CC errors in patients with a propensity to CHO overestimation (i.e., E_3_, E_5_, E_7_ and E_9_) or underestimation (i.e., E_2_, E_4_, E_6_ and E_8_). Table 3 shows the CC error intervals used in each experiment, obtained by adjusting the ∆CHO distribution with a CI of 95%.

E_3_ and E_5_ allowed us to assess the impact of CHO overestimations when the patient performed CC errors within the safe interval with a mean value of −0.5∆CHO_max_ and −0.75∆CHO_max_, respectively. In its turn, E_7_ and E_9_ were used to evaluate the effect of CHO overestimations on the limit and outside the safe interval. Therefore, the ∆CHO distribution used in E_7_ generated a narrow interval of CC errors, centered on −∆CHO_max_, while E_9_ used an interval where 97.5% of the CC errors were outside the safe interval, up to −1.5∆CHO_max_.

Similarly, E_2_ and E_4_ let us evaluate the consequences of CHO underestimations within the safe interval with a mean value of 0.5∆CHO_max_ and 0.75∆CHO_max_, respectively. Finally, E_6_ allowed us to assess the impact of CHO underestimations on the limit and E_8_ outside the safe interval.

**Figure 1:**
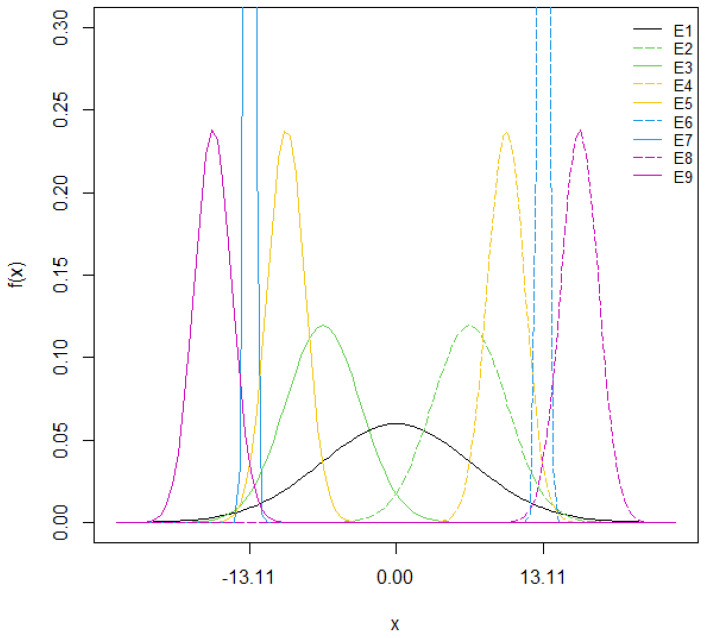
Graphical representation of the normal distribution of CC errors in each experiment for the virtual patient Adult#1, whose ∆CHO_max_ = 13.11 g. Experiments with symmetric error intervals are represented with the same color on the graph. The single lines correspond to the overestimation zone and the dashed lines to the underestimation zone.

As an example, Figure 1 illustrates the ∆CHO distributions applied to create the experiments used to assess the impact of different CC errors on the postprandial BG of the virtual subject Adult#1, whose ∆CHO_max_ = 13.11 g.

### 2.3. Statistical Method

The metrics selected to assess the glycemic outcome after applying the proposed method were as follows: (1) time in range (TIR), defined as the percentage of time spent within the range of 70–180 mg/dL; (2) time above range (TAR), i.e., hyperglycemia, defined as the percentage of time spent above 180 mg/dL; (3) time below range (TBR), i.e., hypoglycemia, defined as the percentage of time spent below 70 mg/dL; (4) the percentage of time spent below 50 mg/dL; and (5) the percentage of time spent above 300 mg/dL.

A normal Q-Q plot and Shapiro–Wilk tests were applied to verify if the percentage values obtained in the experiments followed the normal distribution. As the data did not follow the normal distribution, we decided to use the bootstrap method instead of the central limit theorem because of the small number of patients in the study. Moreover, CIs and hypothesis tests for the difference between the means, using the nonparametric bootstrap method, were used to compare the results of each experiment (E_1_, E_n_, …, E_9_) with the results of the error-free scenario (i.e., E_0_) obtained in each metric. We also define as subjects’ exclusion criteria a TIR < 90% in E_0_ to avoid effects on glucose regulation unrelated to the CHO estimate.

## 3. Results

**Figure 2:**
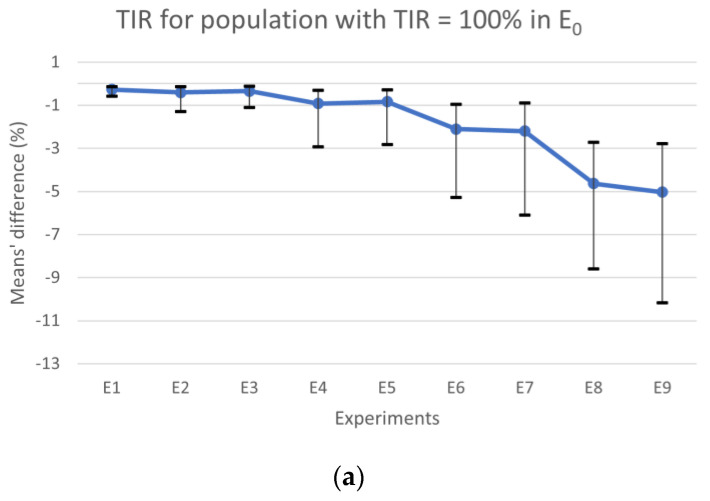

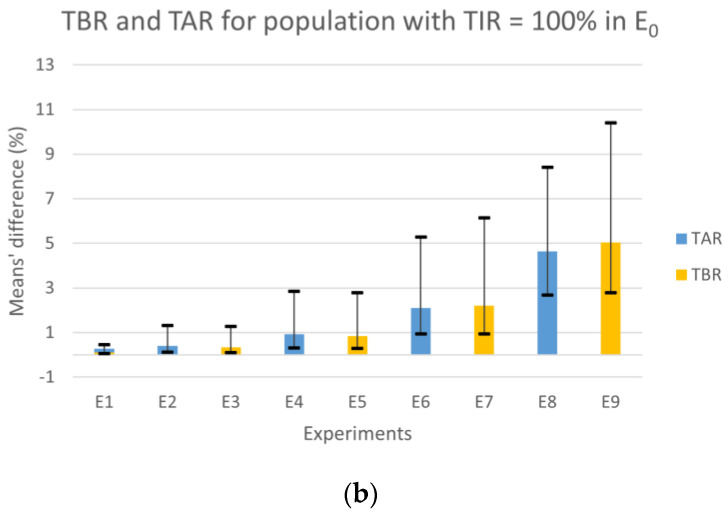
Plots of the difference between the experiments with error and E0 in CC for patients with TIR = 100% (in E_0_), showing the differences in TIR in (**a**) and TBR (yellow bars) and TAR (blue bars) in (**b**). In both plots, the vertical lines represent the CIs. TIR, time in range; TAR, time above range; TBR, time below range; E_0_, error-free experiment.

**Figure 3:**
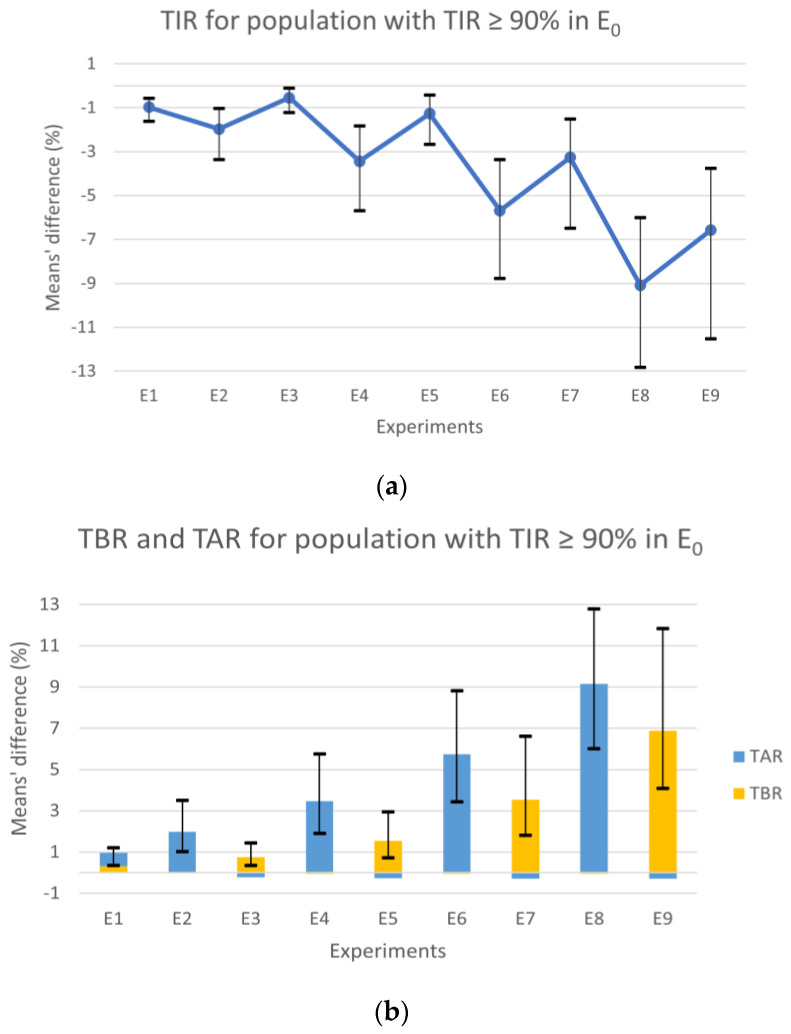
Plots of the difference between the experiments with error and E_0_ in CC for patients with TIR ≥ 90% (in E_0_). (**a**) shows the differences in TIR and (**b**) in TBR (yellow bars) and TAR (blue bars). In both plots, the vertical lines represent the CIs. TIR, time in range; TAR, time above range; TBR, time below range; E_0_, error-free experiment.

After conducting the E_0_ scenario, we found that five patients had a TIR below 90%, so we excluded them from the statistical analysis to avoid potential bias. To further explore the results, we analyzed the data separately for patients who maintained their glucose levels within range throughout the study, i.e., TIR = 100%, and those with a TIR above 90%. We used paired samples to compare the means of these two populations with the means obtained from E_0_ and applied confidence intervals and hypothesis tests. The results for the population with a TIR = 100% and TIR ≥ 90% are summarized in Figure 2 and Figure 3, respectively. Additionally, the tables in Appendix A provide more detailed information on the results in Figure 2 and Figure 3.

Figure 2a shows the results for TIR, the horizontal line represents the experiments’ mean values, and the vertical lines are the CIs. The results obtained for TBR and TAR means’ differences are plotted in Figure 2b with colored bars, whereas the CIs for the most expressive condition (TBR or TAR) in each experiment are represented by vertical lines. When comparing the results obtained from E_0_ with those obtained from experiments where the CC error falls within the range of [−∆CHO_max_, ∆CHO_max_] (i.e., E_1_, E_2_, E_3_, E_4_, and E_5_) for the population with TIR = 100%, we observed that the absolute maximum of the differences between means for TBR, TIR, and TAR were 0.84%, 0.92%, and 0.92%, respectively. Additionally, the differences’ absolute maximum in the CIs for TBR, TIR, and TAR were 2.79%, 2.93%, and 2.85%, respectively. However, for experiments falling outside this interval (i.e., E_6_, E_7_, E_8_ and E_9_), we observed a larger absolute maximum for differences in TBR, which was 5.04%, in TIR was 5.03%, and 4.64% in TAR. In this case, the absolute maximum in the CIs for TBR, TIR, and TAR was 10.40%, 10.17%, and 8.41%, respectively, when compared to E_0_. According to the guidelines, the maximum time spent in hypoglycemia < 50 mg/dL should be less than 1% and generally, for TBR, it should be less than 4% [3]. Therefore, when comparing E_9_ with E_0_, we found that the mean difference for time spent in hypoglycemia < 50 mg/dL was 0.95%, which is close to the limit. Additionally, the TBR was 5.04%, which exceeds the recommended limit. 

After analyzing the results of the population with a TIR of 100%, a second analysis was conducted that included patients with a TIR ≥ 90% (Figure 3), attempting to recreate a more realistic scenario with subjects that can still be considered controlled.

Observing the population with TIR above 90% in E_0_, the experiments’ results for the TIR (Figure 3a) showed a consistent degradation through the experiments with the increase in the error, similar to the results for the population always in range. However, contrary to the results from the first analysis (Figure 2a), the symmetric experiments revealed notable differences, with the scenarios in the underestimation zone showing worse results. The observed decrease in TIR is directly related to the TAR and TBR variations with slight differences. Analyzing Figure 3b, the different deterioration proportions of TAR and TBR in the experiments in under- and overestimation zones, respectively, are also evident. The experiments in the overestimation zone also show a small improvement in TAR, expressed through the short blue bars under zero. The experiments in the underestimation zone showed minor improvements in TBR with values up to one-hundredth.

The experiments E_1_, E_2_, E_3_, and E_4_ continued to show results similar to the error-free scenario, with mean values under 4%. Although, the difference absolute maximum for the CI of E_4_ was 5.76% in the TAR, which is further within the goals of 25% for TAR [3]. In E_6_ and E_8_, a significant deterioration in the TAR compared to the control scenario was observed, reaching a difference of 12.78% in E_8_. Similarly, the symmetrical experiments E_7_ and E_9_ showed an increase in TBR, though it was considerably less. Globally, we found that the results of the experiments where the tendency was to underestimate CHO, that is, where hyperglycemia was more likely to occur, showed less favorable results (Figure 3b blue bars). This fact can be explained by postprandial glycemic peaks that typically happen 1–2 h after a meal [21] since Equation (2) was obtained with the condition that the BG will be close to the target 2–3 h after a meal and not before. 

All the results showed an increase in the CIs as the scenarios included a bigger error. Generally, we could observe a pronounced deterioration in the results in the last four experiments (E_6–9_), where 50 and 97.5% of the meals had a CC error outside the range of [−∆CHO_max_, ∆CHO_max_].

## 4. Discussion

After calculating the maximum allowable error in CHO counting for each patient, such that it did not lead to undesirable postprandial BG fluctuations, we determined that the average value among the 33 subjects in the sample was 9.13 g. This value is in line with the findings of Smart [18], which concluded that errors up to 10 g were considered safe. However, using the ICR and ISF values of each subject to compute the error threshold showed that it could vary greatly. For example, Adolescent#5 presented a maximum error of 5.50 g, while Adolescent#7 showed a much higher maximum value of 16.55 g.

The experiments conducted included a control scenario and nine scenarios introducing different CC errors. A scenario was created in which patients made errors up to the maximum error of both underestimating and overestimating the amount of CHO in the meal, and the remaining scenarios were created to represent situations in which the patient’s tendency was always to underestimate or overestimate. We considered it relevant to perform polarized experiments, as some aforementioned studies [11,14,16,17] demonstrate how the most common tendency is to underestimate larger meals, in contrast to snacks, which are typically overestimated.

It is also important to note that CHO is not the only factor that influences postprandial glycemia, other nutrients present in the meal, such as fats and proteins, as well as previous physical activity, can affect postprandial BG. Thus, two groups of patients were defined for the analysis of results, one with TIR of 100% in the error-free experiment and another for subjects with TIR above 90%. For the group of patients always in range, we could better isolate the impact of incorrect CHO estimations. Nevertheless, having BG always in range is not the most common glycemic profile for patients with T1DM; for that reason, we analyzed the second group with TIR above 90%. This way, we intended to reproduce a more realistic scenario.

The data from the two groups showed some differences. As expected, the glycemic control of the group with TIR greater than 90% deteriorated more sharply, especially in the underestimation zone experiments. This may be due to the postprandial peaks, once the calculation of the maximum error was developed to make BG on target 2–3 h after the meal and not before. In this trial, the patients used continuous glucose monitors to collect BG levels every 5 min, including the peaks logs. It is noteworthy that the goals for T1DM are far more permissive for TAR (<25% of the time) than for TBR (<4%) [3].

To the best of our knowledge, this was the first study to utilize a patient-specific calculation to compute the maximum error in CC. The results demonstrated how the TIR, TAR, and TBR values deteriorated as the error introduced approached the limit, highlighting the last experiments (E_6–9_), where the maximum error was exceeded in 50 and 97.5% of the meals. This shows that the proposed method for defining personalized limits for CC error could be suitable for setting up a new approach to train and evaluate patients’ CC abilities. This method has the potential to personalize treatment and empower the patient by providing them with an understanding of their true limits.

However, it is necessary to note the limitations of this study. The sample used was small, with fewer than 30 subjects remaining for statistical analysis. To overcome this, the nonparametric bootstrap method was implemented. Furthermore, as mentioned in the previous section, the method used to calculate the safe interval was designed to make the postprandial glycemic on target 2–3 h after the meal, leading to an increase in the TAR and, consequently, to the worst TIR values in the scenarios where the patients underestimated CHO. To overcome this, the mathematical model could be adjusted by applying correction factors to the safe interval to compensate for the postmeal peak effect. Although the simulator is an excellent tool for an initial phase of preclinical testing, the study must be repeated in a sample of patients in a real-world context to validate the method. It may also be pertinent to carry out a differentiated analysis for the different age groups.

## 5. Conclusions

From this virtual clinical trial, we concluded that there was a difference in TIR, TAR, and TBR between the experiments with errors in CC and the control experiment. We observed that this difference rose with the increase in the error added, degrading more markedly when approaching the calculated limit in over- and underestimation (experiments E_6–9_). Therefore, the method proposed in [20] allows the establishment of a patient-specific error limit for CC. Given the results of this study, we consider the ∆CHO_max_ an excellent starting point for an initial assessment of patients’ capabilities in CC and to provide appropriate ongoing education. However, it is necessary to repeat the study on actual patients to corroborate these results.

## Figures and Tables

**Table 1:** ∆CHO_max_ obtained for each patient.

ΔCHO_max_ (g)			
	Adults	Adolescents	Children
1	13.11	9.90	5.48
2	16.02	7.72	8.03
3	12.42	12.76	9.58
4	11.11	6.42	5.37
5	8.41	5.50	6.87
6	10.42	10.06	7.40
7	12.78	16.55	5.55
8	7.12	7.04	5.70
9	11.09	7.95	5.89
10	10.46	8.52	10.06
Avg.	11.03	8.63	6.41

ΔCHO_max_ was calculated according to Equation (2) with G_L_, G_T_, and G_H_ defined as 70, 100, and 180 mg/dL, respectively.

**Table 2:** Schedule of patients’ daily meals with the corresponding carbohydrate content.

		CHO_True_ (g)		
	Day Time	Adult	Adolescent	Child
1st meal	08:00 a.m.	30	30	30
2nd meal	12:00 p.m.	45	45	30
3rd meal	04:00 p.m.	30	45	30
4th meal	08:00 p.m.	45	45	30
5th meal	12:00 a.m.	15	30	15

The meal plan was designed based on the methodology described in [8]. CHO_True_, actual carbohydrate content in the meal; g, grams.

**Table 3:** Carbohydrate counting error intervals introduced in each experiment.

Experiment	CC Error Interval
E_0_	-
E_1_	$\left[-{∆CHO}_{max},\;{∆CHO}_{max}\right]$
E_2_	$\left[0,\;{∆CHO}_{max}\right]$
E_3_	$\left[-{∆CHO}_{max},\;0\right]$
E_4_	$\left[0.5\;{∆CHO}_{max},\;{∆CHO}_{max}\right]$
E_5_	$\left[-{∆CHO}_{max},\;{-0.5∆CHO}_{max}\right]$
E_6_	$\left[0.95\;{∆CHO}_{max},\;{1.05∆CHO}_{max}\right]$
E_7_	$\left[-{1.05∆CHO}_{max},\;-0.95\;{∆CHO}_{max}\right]$
E_8_	$\left[{∆CHO}_{max},\;{1.5∆CHO}_{max}\right]$
E_9_	$\left[-{1.5∆CHO}_{max},\;-{∆CHO}_{max}\right]$

All the intervals present a confidence interval of 95%. ΔCHO_max_: absolute maximum error allowed in carbohydrate counting.

## Data Availability

The data that support the findings of this study are available from the corresponding author upon reasonable request.

## Appendix A

The tables included in this section provide the obtained results in detail, displaying mean values along with confidence interval estimates.

**Table A1:** Confidence intervals for the difference between two population means (paired samples) for patients with TIR = 100% in E_0_.

		BG < 50 (%)	BG < 70 (%)	TIR (%)	BG > 180 (%)	BG > 300 (%)
E_1_	Mean	0.0032	0.11	−0.27	0.16	0.00
	CI	(0.00, 0.0079)	(0.043, 0.23)	(−0.57, −0.14)	(0.059, 0.46)	(0.00, 0.00)
E_2_	Mean	0.00	0.00	−0.41	0.41	0.00
	CI	(0.00, 0.00)	(0.00, 0.00)	(−1.30, −0.13)	(0.13, 1.32)	(0.00, 0.00)
E_3_	Mean	0.0063	0.34	−0.34	0.00	0.00
	CI	(0.00, 0.019)	(0.11, 1.28)	(−1.11, −0.11)	(0.00, 0.00)	(0.00, 0.00)
E_4_	Mean	0.00	0.00	−0.92	0.92	0.00
	CI	(0.00, 0.00)	(0.00, 0.00)	(−2.93, −0.31)	(0.31, 2.85)	(0.00, 0.00)
E_5_	Mean	0.045	0.84	−0.84	0.00	0.00
	CI	(0.00, 0.14)	(0.28, 2.79)	(−2.82, −0.29)	(0.00, 0.00)	(0.00, 0.00)
E_6_	Mean	0.00	0.00	−2.11	2.10	0.00
	CI	(0.00, 0.00)	(0.00, 0.00)	(−5.29, −0.95)	(0.93, 5.28)	(0.00, 0.00)
E_7_	Mean	0.38	2.20	−2.20	0.00	0.00
	CI	(0.00, 1.60)	(0.93, 6.14)	(−6.09, −0.90)	(0.00, 0.00)	(0.00, 0.00)
E_8_	Mean	0.00	0.00	−4.64	4.64	0.00
	CI	(0.00, 0.00)	(0.00, 0.00)	(−8.59, −2.71)	(2.68, 8.41)	(0.00, 0.00)
E_9_	Mean	0.95	5.04	−5.03	0.00	0.00
	CI	(0.043, 4.88)	(2.79, 10.40)	(−10.17, −2.78)	(0.00, 0.00)	(0.00, 0.00)

All the results are expressed with two significant figures. BG, blood glucose; TIR, time in range; CI, confidence interval.

**Table A2:** Confidence intervals for the difference between two population means (paired samples) for patients with TIR ≥ 90% in E_0_.

		BG < 50 (%)	BG < 70 (%)	TIR (%)	BG > 180 (%)	BG > 300 (%)
E_1_	Mean	0.068	0.32	−0.97	0.65	0.00
	CI	(0.0032, 0.32)	(0.15, 0.75)	(−1.63, −0.57)	(0.35, 1.21)	(0.00, 0.00)
E_2_	Mean	0.0089	−0.021	−1.97	1.99	0.00
	CI	(0.00, 0.036)	(−0.11, 0.00)	(−3.36, −1.03)	(1.03, 3.51)	(0.00, 0.00)
E_3_	Mean	0.049	0.75	−0.54	−0.22	0.00
	CI	(0.0036, 0.22)	(0.35, 1.44)	(−1.22, −0.10)	(−0.59, −0.061)	(0.00, 0.00)
E_4_	Mean	−0.0014	−0.061	−3.44	3.47	0.00
	CI	(−0.010, 0.00)	(−0.19, −0.011)	(−5.69, −1.83)	(1.90, 5.76)	(0.00, 0.00)
E_5_	Mean	0.11	1.54	−1.27	−0.27	0.00
	CI	(0.021, 0.37)	(0.73, 2.95)	(−2.66, −0.43)	(−0.78, −0.071)	(0.00, 0.00)
E_6_	Mean	−0.0028	−0.065	−5.69	5.74	0.00
	CI	(−0.020, 0.00)	(−0.21, −0.011)	(−8.77, −3.36)	(3.43, 8.82)	(0.00, 0.00)
E_7_	Mean	0.43	3.54	−3.26	−0.28	0.00
	CI	(0.067, 1.41)	(1.82, 6.61)	(−6.48, −1.51)	(−0.80, −0.069)	(0.00, 0.00)
E_8_	Mean	0.0014	−0.050	−9.09	9.16	0.00
	CI	(0.00, 0.0043)	(−0.14, −0.011)	(−12.83, −6.00)	(6.02, 12.78)	(0.00, 0.00)
E_9_	Mean	1.16	6.87	−6.58	−0.28	0.00
	CI	(0.36, 3.49)	(4.09, 11.84)	(−11.52, −3.77)	(−0.79, −0.069)	(0.00, 0.00)

All the results are expressed with two significant figures. BG, blood glucose; TIR, time in range; CI, confidence interval.

## References

[1] Roep B.O., Thomaidou S., van Tienhoven R., Zaldumbide A. (2020). Type 1 diabetes mellitus as a disease of the β-cell (do not blame the immune system?). Nat. Rev. Endocrinol..

[2] Wang Y., Zhang J., Zeng F., Wang N., Chen X., Zhang B., Zhao D., Yang W., Cobelli C. (2017). “Learning” Can Improve the Blood Glucose Control Performance for Type 1 Diabetes Mellitus. Diabetes Technol. Ther..

[3] ElSayed N.A., Aleppo G., Aroda V.R., Bannuru R.R., Brown F.M., Bruemmer D., Collins B.S., Hilliard M.E., Isaacs D., Johnson E.L. (2022). 6. Glycemic Targets: Standards of Care in Diabetes—2023. Diabetes Care.

[4] Ratner R.E. (2018). Hypoglycemia: New Definitions and Regulatory Implications. Diabetes Technol. Ther..

[5] Rodriguez H., El-Osta A. (2018). Epigenetic Contribution to the Development and Progression of Vascular Diabetic Complications. Antioxid. Redox Signal..

[6] American Diabetes Association (2014). Diagnosis and Classification of Diabetes Mellitus. Diabetes Care.

[7] ElSayed N.A., Aleppo G., Aroda V.R., Bannuru R.R., Brown F.M., Bruemmer D., Collins B.S., Hilliard M.E., Isaacs D., Johnson E.L. (2022). 9. Pharmacologic Approaches to Glycemic Treatment: Standards of Care in Diabetes—2023. Diabetes Care.

[8] Abreu C., Miranda F., Felgueiras P. (2022). Towards patient-specific carbohydrate counting accuracy: An in silico study. AIP Conference Proceedings.

[9] Deeb A., Al Hajeri A., Alhmoudi I., Nagelkerke N. (2016). Accurate Carbohydrate Counting Is an Important Determinant of Postprandial Glycemia in Children and Adolescents With Type 1 Diabetes on Insulin Pump Therapy. J. Diabetes Sci. Technol..

[10] Vaz E.C., Porfírio G.J.M., Nunes H.R.d.C., Nunes-Nogueira V.d.S. (2018). Effectiveness and safety of carbohydrate counting in the management of adult patients with type 1 diabetes mellitus: A systematic review and meta-analysis. Arch. Endocrinol. Metab..

[11] Kawamura T., Takamura C., Hirose M., Hashimoto T., Higashide T., Kashihara Y., Hashimura K., Shintaku H. (2015). The factors affecting on estimation of carbohydrate content of meals in carbohydrate counting. Clin. Pediatr. Endocrinol..

[12] Brazeau A., Mircescu H., Desjardins K., Leroux C., Strychar I., Ekoé J., Rabasa-Lhoret R. (2012). Carbohydrate counting accuracy and blood glucose variability in adults with type 1 diabetes. Diabetes Res. Clin. Pract..

[13] Meade L.T., Rushton W.E. (2016). Accuracy of Carbohydrate Counting in Adults. Clin. Diabetes.

[14] Reiterer F., Freckmann G., del Re L. (2018). Impact of Carbohydrate Counting Errors on Glycemic Control in Type 1 Diabetes. IFAC-PapersOnLine.

[15] Gurnani M., Pais V., Cordeiro K., Steele S., Chen S., Hamilton J.K. (2018). One potato, two potato, … assessing carbohydrate counting accuracy in adolescents with type 1 diabetes. Pediatr. Diabetes.

[16] Roversi C., Vettoretti M., Del Favero S., Facchinetti A., Sparacino G. (2020). Modeling Carbohydrate Counting Error in Type 1 Diabetes Management. Diabetes Technol. Ther..

[17] Smart C.E., Ross K., Edge J.A., King B.R., McElduff P., Collins C.E. (2010). Can children with Type 1 diabetes and their caregivers estimate the carbohydrate content of meals and snacks?. Diabet. Med..

[18] Smart C.E., Ross K., Edge J.A., Collins C.E., Colyvas K., King B.R. (2009). Children and adolescents on intensive insulin therapy maintain postprandial glycaemic control without precise carbohydrate counting. Diabet. Med..

[19] Smart C.E., King B.R., McElduff P., Collins C.E. (2012). In children using intensive insulin therapy, a 20-g variation in carbohydrate amount significantly impacts on postprandial glycaemia. Diabet. Med..

[20] Abreu C., Miranda F., Felgueiras P. (2018). Carbohydrate counting: How accurate should it be to achieve glycemic control in patients on intensive insulin regimens?. AIP Conference Proceedings.

[21] Daenen S., Sola-Gazagnes A., M’bemba J., Dorange-Breillard C., Defer F., Elgrably F., Larger É., Slama G. (2010). Peak-time determination of post-meal glucose excursions in insulin-treated diabetic patients. Diabetes Metab..

